# Diagnosis Protocol of Stomach Distemperament for Clinical Practice in Iranian Traditional Medicine: A Narrative Review

**Published:** 2017-07

**Authors:** Mahdi ALIZADEH, Ebrahim KHADEM, Jale ALIASL

**Affiliations:** 1. Dept. of Iranian Traditional Medicine, School of Iranian Traditional Medicine, Tehran University of Medical Sciences, Tehran, Iran; 2. Research Institute for Islamic and Complementary Medicine, School of Iranian Traditional Medicine, Iran University of Medical Sciences, Tehran, Iran

**Keywords:** Diagnostic protocols, Stomach, Distemperament, Iranian traditional medicine

## Abstract

**Background:**

In Iranian traditional medicine (ITM) stomach is the important organ in the body. Its disorders can affect other organs such as liver, heart and also can cause depression. Stomach distemperaments can cause some disorders. The purpose of this study was to provide a diagnostic method of stomach distemperament for clinical practice.

**Methods:**

In this study authoritative ITM books such as Canon of Avicenna, Zakhirah-E-Kharazm Shahi by Hakim Esmail Jorjani, *Kamel al*-Sina’ah al-Tibbiyah by Ali ibn al-‘Abbas al-Majusi were assessed and symptoms and signs of stomach distemperament were collected.

**Results:**

Stomach has some normal temperament. The imbalance in temperament and humor can cause distemperments. There are 12 types of stomach distemperament and based on symptoms and signs a primary protocol is designed for diagnosis of stomach distemperament.

**Conclusion:**

There is no available guideline for gastric distemperamet diagnosis protocol. As a result, the presented protocol should be considered for use in clinical practice.

## Introduction

The stomach is an important organ in the body and it plays a vital role in digestion of foods (1–3). GI diseases are a source of substantial morbidity, mortality, and cost in the United States (4). In Iranian traditional medicine (ITM) stomach is very important organ and its disorders can affect other organs such as liver, heart and can cause depression (1, 5–9).

In ITM, physiological functions of the human body are considered to be based on main factor, known as Temperament [Mizaj] and humor (3, 10). The Arabic meaning of temperament (mizaj) is the “qualitative mixture” built from the elements (warm, cold, wet or damp, and dry and their combinations (2, 3). In ITM, Mizaj (temperament) plays a key role in preventive, therapeutic, and lifestyle recommendations (1–3). Normally there are four senses of humor in the human body: Phlegm or Balgham, Blood or Dam, Yellow bile or Safra, and Black bile or Sauda (1–3). Their imbalances in the body cause some disorders (1–3). Each organ in the body has a normal and special mizaj. For example, brain is cold and wet, heart is warm and dry and stomach has some normal types of temperaments based on Canon of Avicenna (2, 3, 11).

Normal stomach temperaments include warm, cold, dry, wet and their combinations. Warm normal stomach temperament has good digestion of dense foods like beef and light foods spoilage such as the chicken and milk, and good digestion but less appetite. In normal cold stomach temperament, appetite is normal but digestion is weak and does not digest the food, except the gentle light. Normal dry temperament has frequent thirsty in the habit, and thirst is eliminated by little drinking and stomach fullness occurs with more amounts of water, and finally, normal wet temperament has less thirst but bears more amount of fluid without any fullness sensation (2). According to signs and symptoms, stomach temperaments are classified into normal and abnormal types. The disease state starts by distemperament (Sue-mizaj), which is a change in the normal temperament of an individual, or of an organ, to a new temperament that is outside the range of normal. Thus, the state of disease in ITM is based on distemperament. There are 12 types of stomach distemperament (2, 3). Distemperant of stomach can cause some disorders such as weak digestion, gastric pain, and gastric irritation (12, 13), primary bile reflux gastritis (14) and gastric ulcer (15). Early detection of stomach distemperments and their treatment can prevent the occurrence of these diseases. Unfortunately, there is not any standard diagnostic protocol for diagnosis of stomach distemperment. The purpose of this study was to provide a diagnostic protocol of stomach distemperament for clinical practice.

## Methods

Authoritative ITM books such as Canon of Avicenna (Ibn Sina) (16), Zakhirah -E-Kharazm Shahi by Hakim Esmail Jorjani (3), *Kamel al*-Sina’ah al-Tibbiyah by Ali ibn al-‘Abbas al-Majusi (17), Sharh ol Asbab Va Alamat by Hakim Nafis ibn Avaz-e-kerman (18), Exire Azam by Hakim Azam Khan cheshti (1), and Alhavi by Muhammad ibn-Zakariya al-Razi (19), Kholasat-al-hekma by Aghili Khorasani (20), were assessed and symptoms and signs of stomach distemperament were collected and based on the repetition in books and expert opinion, clinical significance and pathognomonic symptoms and signs were classified in major and minor criterias and an appendix.

## Results

Stomach distemperaments, based on signs and symptoms are classified into different types; with or without humeric substance. In this study, signs, and symptoms are classified in major and minor criteria for diagnosing the stomach distemperaments (Table 1, 2).

**Table 1:** Major (principal) criteria for stomach distemperament type assessment

**Symptoms**	**Stomach distemperament Types**							
	**Warm**	**Cold**	**Wet**	**Dry**	**Choleric**	**Warm-wet with wet substance**	**Phlegmatic**	**Melancholic**
**Digestion**	**Strong**	**Weak**	**Weak**	**Weak**	**Strong**	*	**Weak**	**Weak**
Appétit	Decreased (sometimes with impatience on hunger)	Increased	*^1^	*	Mostly decreased^2^ Increased in extreme conditions	Moderate	Decreased (Increased in sour Phlegm)	Increased
Thirstiness	+	−	−	++ sometimes with ***Takhazkhoz*** (splash sound) after drinking)	+ (for cold water)	+ (without bitter or salty sensation in the mouth)	−/false +^3^	*
Transition time	Rapid	Slow	Rapid	Slow	*	Rapid	*	Slow
Stool consistency	*	Wet	Wet	Dry	*	*	*	*
Tendency to	Bad and stinky substance, Mobaredat^4^	Mosakhenat	*	*	*	*	Hot Spice, and in viscous **Phlegm** tendency to Salty, spicy and sour tastes	*
Hatred of	Mosakhenat	Mobaredat	Wet and creamy	Yabese ^5^	*	*	Abundant amount of food intake	*
Benefit from	Mobaredat	Hot Spice and administration of warm things on stomach	Low amount food intake /Yabese	Wet climate, Moratebat, meat soup, Oils	*	*	Administration of warm things on stomach cardia	*
Disadvantage of	Mosakhenat	*	Moratebat, creamy and cold water drinking and wet fruits and legume	*	*	*	Hot Spice	*

1 * there not found any documents in Text books in this factor /

2 (sometimes with Impatience on hunger) /

3 Salty or viscous Phlegm which causes nausea and relives with warm water /

4 tempramnt cooler substance like kampher /

5 tempramnt drier substance

**Table 2:** Minor criteria (Appendix) for stomach distemperament type's assessment

**Other Symptoms**		**Stomach distemperament Types**									

		**Warm**	**Cold**	**Wet**	**Dry**	**Choleric**	**Warm-wet with wet substance**	**Phlegmatic**	**Melancholic**
Other gastrointestinal Symptoms	Stomach discomfort	+( Relief with Mobaredat)	*	**+/− (** In case of Chronicity )		*	*	*	+ (Especially before meals)
	Burning sensation in stomach	*	•	*	*	**+**	*	+(sour, salty)	+( Relieved by eating)
	Hot sensation in the stomach	*	*	*		**+**	**+**	+( Salty)	*
	Regurgitation	*	*	*	*	*	**+**	*	*
	Fasciculation of abdominal wall	*	*	*	*	*	*	*	**+**
	Nausea & vomiting	*	*	**+**	*	**+**	**+**	+	+(sour Mozaras
	Stool	Merari	Indigested	*	*	Choleric / Merari	*	Phlegm	*
	Taste & smell	Taste of rotten fish	Sour (low cold)	*	*	Bitterness	Greacy sensation in the Mouth	Sour, insipidous (insipidous Phlegm) salty (salty Phlegm)	Sour tooth sensibility (Before meals)
	Saliva	Oral temperature	*	Excessive and Foam	Excessive dryness of the mouth and tongue and a little saliva	*	Increased during starvation	Increase (while sleeping)	Dry mouth
Body	Tongue color	*	*	White and milky	*	*	Redness and roughness	[White and milky]	Black
	Physique	*	*	Flabby	Skinny and Cachexia	Skinny and Cachexia	*	Flabby	[Slimness and Cachexia]
	Color	*	Yellow / White	*	*	*	*	White	Yellow / Black
	Pulse	*	Slow	Softness	*	*	*	*	Small / frequency
	Other possible diseases	*	*	Dropsy	*	*	*	Dropsy	Splenomegaly /fear & obsession/ (hemorrhoids & varicose veins)

## Discussion

Temperament has an important role in human body functions (21). According to ITM, stomachs have different temperament types. Each temperament type is susceptible to certain diseases (1–3). Imbalance of humors and mizaj in stomach is known as distemperments (1–3). Distemperments of stomach can causes symptoms such as bloating (22) belching, stomach pain (12, 23) heartburn and reflux (24). Diagnosis of distemperament can help us to predict susceptibility to some diseases such as peptic ulcer (15), IBS (23) and fatty liver (8) before the onset of illnesses. Melancholic stomach distemperament can lead stomach cancer (1–3). Therefore, with diagnosis of stomach distemperament we can decrease predisposition of diseases, illnesses, and reverse pathological process. In fact, these disorders may be prevented by suitable nutrition and lifestyle (25) and decrease the cost of diseases such as heartburn, reflux and peptic ulcer. It is clear the early diagnosis of stomach distemperment is very important, however, until now; there is no available guideline for gastric distemperamet diagnosis, therefore, this protocol could be used for early diagnosis of stomach distemperment. However, the cross-sectional study was found for assessment of mizaj (temperament). They designed a questionnaire based on the mizaj identification in ITM text books -with 52 item and assessed its reliability and variability (26).

The present protocol should be considered for use in clinical practice. Iranian Traditional Medicine Gastroenterology Research Committee of Khark (ITM-GRCK) will examine the reliability and validity of theses protocol in further studies and the results will be published in near future.

## Conclusion

Diagnose of stomach distemperament is very important in Iranian Traditional Medicine.

The present protocol is suggested for primary diagnose of stomach distemperament in clinical practice.

## Ethical considerations

Ethical issues (Including plagiarism, informed consent, misconduct, data fabrication and/or falsification, double publication and/or submission, redundancy, etc.) have been completely observed by the authors.

## Appendix: (digestion status evaluation)

**Table:**

**Specific digestion questions**		**Stomach distemperament Types**					

		**Warm**	**Cold**	**Wet**	**Choleric**	**Phlegmatic**	**Melancholic**
Signs of weak digestion	Stomach fullness 3 h after meal	*	+	*	*	*	*
	Belching 3 h after meal	Smoky/sour	Sour	*	Smoky/stinky	Sour	*
	Gastric distension 3 h after meal	*	**+**	*	*	*	*
	Stomachache 3 h after meal	*	**+**	*	*	*	*
	Bloating in stomach	*	**+**	*	*	*	**++**
	Rumbling, growling stomach	*	**+**	*	*	*	*
	Postprandial sleepiness, dizziness, headache or vertigo	*	*	+	*	*	*
	Decrease of bloating, distension or nausea with belching	*	*	*	*	**+**	*
	Sour taste sensation in starvation or after meal	*	Postprandial	*	*	*	In hunger
Signs of strong digestion	Easy digestion of dense or cold foods	**+**	*	*	*	*	*
	Rapid Spoilage of Soft, low amount and warm temperate substance	**+**	*	*	*	*	*
